# Letter from the Editor in Chief

**DOI:** 10.19102/icrm.2021.120907

**Published:** 2021-09-15

**Authors:** Moussa Mansour


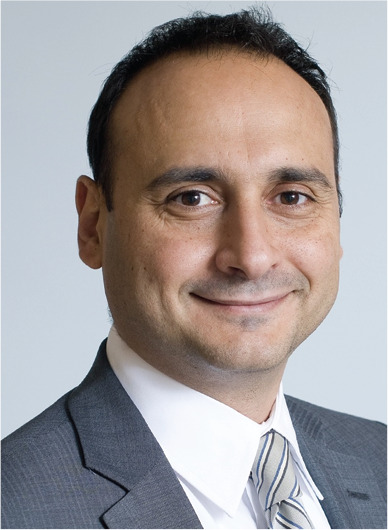


Dear Readers,

In this issue of *The Journal of Innovations in Cardiac Rhythm Management*, I would like to highlight the publication titled “Use of Stereotactic Radioablation Therapy as a Bailout Therapy for Refractory Ventricular Tachycardia in a Patient with a No-entry Left Ventricle” by Aras et al., which documents the case of a patient with double mechanical valves in the aortic and mitral positions who was treated for refractory ventricular tachycardia (VT) using noninvasive stereotactic radioablation.

Interest in noninvasive ablation as a therapeutic option has grown since a first-in-man series was published in 2017,^1^ and many small studies have reported success in treating VT refractory to conventional treatment approaches, such as medications and catheter-based ablation, using this technique. There are, however, significant challenges associated with the use of noninvasive radioablation, including accurate target identification, the need to compensate for respiratory motion, and the occurrence of adverse events. Some side effects related to radiation toxicity can occur early and include pericarditis, pneumonitis, and the exacerbation of heart failure, while others may occur late and include pericardial fistula, pericardial effusion, and worsening of mitral regurgitation.

To date, most published studies on noninvasive cardiac ablation have used traditional radiation, where X-rays (photons) are delivered to the target area. Although effective, this approach inadvertently results in the ablation of normal tissue beyond the area of interest, leading to complications. In the field of oncology, a new form of radioablation, proton therapy, has recently been introduced, where protons are directed to the target area and deposit most of their radiation at a more controllable tissue depth, with minimal residual radiation occurring beyond the target. A recent oncology study demonstrated a superior safety profile of proton radiation compared to photon treatment.^2^ There are also preliminary studies that suggest the feasibility of using proton radiation for the treatment of cardiac arrhythmias,^3^ and at least one multicenter study for VT ablation is currently being planned in the United States.

I hope you find this issue of *The Journal of Innovations in Cardiac Rhythm Management* educational.

Sincerely,


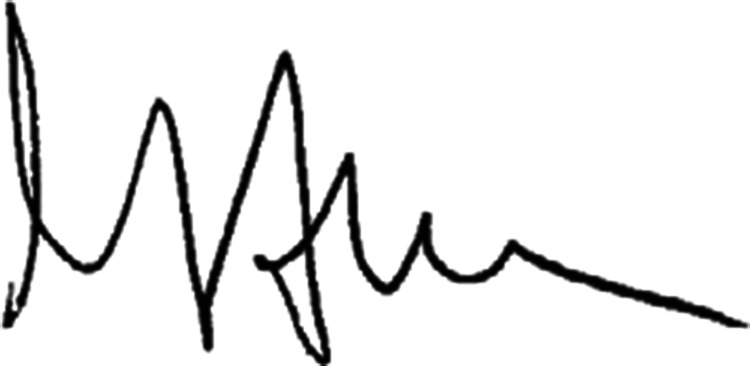


Moussa Mansour, md, fhrs, facc

Editor in Chief

*The Journal of Innovations in Cardiac Rhythm Management*

MMansour@InnovationsInCRM.com

Director, Atrial Fibrillation Program

Jeremy Ruskin and Dan Starks Endowed Chair in Cardiology

Massachusetts General Hospital

Boston, MA 02114
